# Evaluation of Glass Ionomer Cement and Composite Resin Restorations in Hypomineralized Permanent First Molars: A Systematic Review and Meta-Analysis

**DOI:** 10.7759/cureus.81265

**Published:** 2025-03-27

**Authors:** Malayka Shah, Megha C Patel, Foram Patel, Miyola Fernandes, Disha Makwani, Miral Mehta

**Affiliations:** 1 Pedodontics and Preventive Dentistry, Karnavati School of Dentistry, Karnavati University, Gandhinagar, IND; 2 Pedodontics and Preventive Dentistry, Karnavati School Of Dentistry, Karnavati University, Gandhinagar, IND; 3 Dentistry, Gold Dental Studio, Old Goa, IND; 4 Public Health, Asian Development Research Institute, Patna, IND

**Keywords:** composite resin, glass ionomer cement, hypomineralized permanent first molars, molar hypomineralization, molar incisor hypomineralization, restorative treatment

## Abstract

Restorative treatment for molars affected by molar incisor hypomineralization (MIH) presents a challenge due to the altered enamel structure. It is critical to understand the evidence base of the available restorative options, due to the high treatment burden for such teeth. This systematic review and meta-analysis aimed to evaluate and compare the success rates of glass ionomer cement (GIC) and composite resin restorations in hypomineralized first permanent molars and to further evaluate the restorations on the basis of modified United States Public Health Service (USPHS) criteria. This review was registered in International Prospective Register of Systematic Reviews (PROSPERO) database as CRD42024566898.

Searches were conducted in PubMed, Google Scholar and Ebsco from January 2000 to May 2024. A total of 13 studies were included according to the Preferred Reporting Items for Systematic Reviews and Meta-Analysis (PRISMA) guidelines in qualitative synthesis and meta-analysis. The over-all success rate was calculated for each study as the primary outcome. The success rates for different parameters of the modified USPHS criteria was calculated as secondary outcome. The Cochrane Collaboration's tool and risk of bias in non-randomized studies of intervention (ROBINS-I) tool were used to assess the risk of bias. The combined effect, heterogeneity and publication bias were analyzed using Stata 17.0 (StataCorp LLC, College Station, TX, USA). A p-value <0.05 was regarded as statistically significant.

Composite resin restorations presented significantly superior results over GIC for overall success (p = 0.0004), marginal adaptation (p = 0.0124) and surface texture (p <0.0001). For the parameters such as retention (p = 0.06), secondary caries (p = 0.20), marginal discolouration (p = 0.3830) and colour match (p = 0.1440) composite resin performed better; however, the difference was statistically nonsignificant. Considering the constraints of this systematic review and meta-analysis, it can be recommended that composite resin restorations presented superior results over GIC for hypomineralized permanent first molars. Complete removal of the hypomineralized tissue is recommended.

## Introduction and background

The term 'molar incisor hypomineralization' (MIH) can be defined as a specific form of qualitative enamel hypomineralization caused by the disruptive ameloblastic activity that occurs during the pre-eruptive maturative stages of amelogenesis and affects one or more first permanent molars and/or incisors [1]. The deposited enamel though of normal thickness, is unusually soft, and fragile, has sub-surface porosities, and has increased organic content making it susceptible to post-eruptive breakdown under the influence of the masticatory forces [2]. The etiology of MIH is unknown and is more likely to be attributable to a combination of systemic and environmental factors rather than a single etiological factor. The contributing factors may include maternal conditions during pregnancy, genetic susceptibility, and childhood illness (pyrexia, chicken pox, otitis media, respiratory diseases, etc.) [3]. Studies have demonstrated prevalence of MIH ranging from 2.5% to 40% [1].

Clinically, the teeth affected by MIH show variations in severity. Teeth affected by a mild form of MIH show isolated demarcated opacities that are white to brown in colour usually on the non-stress bearing areas of first permanent molars without tooth structure loss. The teeth affected by moderate and severe forms of MIH show demarcated opacities that involve occlusal or incisal thirds of teeth with signs of post-eruptive breakdown and evidence of widespread caries. Depending on the severity and susceptibility of an individual, the hypomineralization lesions may occur independently or co-exist with the hypoplastic lesions [4]. The teeth affected by MIH are extremely sensitive even to the slightest of stimuli such as brushing, leading to the accumulation of dental plaque, the onset of dental caries, and subsequent exposure of the underlying dentin and pulp [2].

Children’s quality of life is also negatively affected by MIH as they often complain of shooting pain, which is usually stimulated by eating cold food [5]. Factors such as difficulty in anaesthetizing the affected tooth due to subclinical inflammation of the pulp, rapid rate of caries development, extreme sensitivity, limited cooperation of a young child, etc. make the management difficult. Aberrant enamel destruction in MIH affects permanent first molars and causes repeated marginal breakdown and recurrent loss of restorations due to decreased bond strength, significantly contributing to increased treatment costs [2]. The MIH-affected enamel shows crystallites that are loosely packed, disorganized enamel prisms, and have less mineral content and porous structure having a direct correlation with the reduced strength and hardness of enamel. Individuals with teeth affected by MIH undergo treatment 10 times more often than those without MIH [5].

Restorations are required in cases of MIH having post-eruptive enamel breakdown with or without any associated carious lesion. Survival of restorations in teeth affected by MIH is high but it still requires a clinical consensus on better approaches for cavity preparation and restorative protocols [5,6]. Different restorative treatment modalities for MIH include fissure sealants, glass ionomer cement (GIC), composite resin, resin-modified GIC, polyacid-modified composite resin, and indirect alloys. The GICs are thought to be effective anticaries cement as they act as a reservoir of fluoride and other ions in the oral cavity. It provides a long-lasting seal and a mechanical barrier that protects the tooth surface. It can be used both as an intermediate as well as a definitive restorative material. Self-etching enhances the adhesion strength of the composite resin to hypomineralized enamel. The amount of pore depth and cracks increases by acid etching with 37% phosphoric acid in MIH-affected teeth, but it affects the adhesiveness of the restorative material. Universal adhesives have been used as an alternative to 37% phosphoric acid etching in such cases [7]. Because of the reduced mineral content, adhesives have a lesser ability to adhere to the tooth surface. Many ultrastructural and biochemical types of research on MIH-affected teeth showed that full-thickness enamel adjacent to the clinical MIH defect was affected to a lesser degree whereas the underlying dentin didn’t show essential alterations in its structure [8].

Cavity margins are difficult to define in MIH-affected teeth. Cavity design plays a critical role as it determines the success of restoration [9]. Two different approaches have been proposed for the cavity margins. Lygidakis et al. and Fayle et al. suggest the removal of the soft porous enamel surrounding the cavity till the resistance of the bur is felt against the hypomineralized enamel. Whereas, William et al. and Mathu-Maju and Wright recommend the complete removal of the defective enamel such that the cavity margins end in sound enamel increasing the resin retention [10]. Mathu-Maju and Wright also recommend pretreatment of enamel with 5% sodium hypochlorite whereas others recommend removal of the defective hypomineralized enamel before bonding [11].

The GIC and composite resin restorations are the two most common materials used in pediatric dentistry, not only for restoring MIH-affected molars but also as part of routine restorative procedures. Many studies have assessed the clinical success of GIC and composite resin in MIH-affected teeth but they lack consensus regarding the superiority of one material over another. The purpose of the present systematic review and meta-analysis was to describe GIC and composite resin as restorative materials for MIH-affected molars and to evaluate the superiority of one over the other with overall clinical success as the primary outcome and the number of restorations showing the best clinical success, i.e., the number of restorations receiving alpha scores per the modified United States Public Health School (USPHS) criteria as secondary outcome.

## Review

Methodology

Protocol and Registration

This systematic review was registered in the International Prospective Register of Systematic Reviews (PROSPERO) database (CRD42024566898) and conducted per the Preferred Reporting Items for Systematic Reviews and Meta-Analysis (PRISMA) guidelines [12] for randomized clinical trials and Meta-analysis of Observational Studies in Epidemiology (MOOSE) guidelines [13] for observational studies.

Eligibility Criteria 

The following inclusion and exclusion criteria (Table 1) were used for the selected studies. The population, intervention, comparison, outcome, and study design (PICOS) format (Table 2) explored the question ‘Do composite resin restorations show better clinical success compared to glass ionomer cement in hypomineralised first permanent molars?’

**Table 1:** Eligibility criteria MIH: Molar incisor hypomineralization, GIC: Glass ionomer cement

Criteria	Description
Inclusion criteria	Randomized clinical trials, single-arm interventional studies, prospective cohort studies, and retrospective cohort studies. Patients in the age group of six to 16 years having hypomineralized permanent molars that are either restored with GIC or composite resin.
Exclusion criteria	Studies dealing with other treatment options such as non-invasive therapies, desensitization, pit and fissure sealants, ceramic or metal restorations, and/or studies to solely improve the aesthetics of MIH-affected teeth were also excluded. Case reports, case series, in vitro studies, reviews, short communications, surveys, editorials, and letters to the editor were excluded. The language was restricted to English only.

**Table 2:** The PICOS format PICOS: Population, intervention, comparison, outcome, and study design; GIC: Glass ionomer cement

Criteria	Determinants
P (population)	Children having hypomineralised first permanent molars in the age group of six to 16 years
I (intervention)	Use of composite resin
C (comparison)	Use of GIC
O (outcome)	Primary outcome: Overall success of the restorative material; Secondary outcome: Number of restorations showing best clinical success
S (study design)	Randomized clinical trials, single-arm interventional studies, prospective cohort studies and retrospective cohort studies

Search Strategy 

One investigator performed a comprehensive electronic search across three different databases using the search strategy described in Table 3. The databases PubMed, Google Scholar, and Ebsco were meticulously searched for the studies that dealt chiefly with restorative treatment modalities for hypomineralized permanent molars from January 2000 to May 2024. The initial keywords used were ‘hypomineralization,’ ‘hypomineralisation,’ ‘hypocalcification,’ ‘hypomaturation,’ ‘permanent molars,’ ‘treatment,’ ‘therapy,’ ‘restoration,’ and ‘adhesion.’ These keywords were paired with the Boolean operators ‘AND’ or ‘OR’ in an advanced search, along with the medical subject headings (MeSH) terms provided by PubMed, to yield the most relevant results. Separate searches were conducted across various databases to retrieve relevant articles related to each treatment method for hypomineralized first permanent molars.

**Table 3:** Search strategy for each database (May 31, 2024)

Database	Search strategy	No. of studies
PubMed	((hypomin* OR hypocalcifi* OR hypomatur*) OR (MIH OR molar-incisor hypominerali*) OR hypomin* permanent molars) AND (treatment OR therapy OR restoration* OR restorative* OR bond* OR adhesive*)	994
Google Scholar	(("hypomineralisation" OR "hypomineralization" OR "hypomaturation" OR "hypocalcification") OR ("molar incisor hypomineralization" OR "molar incisor hypomineralisation") OR (“hypomineralised permanent molars” OR “hypomineralized permanent molars”)) AND ("restoration" OR "restorative" OR "adhesion" OR "adhesive" OR "bond")	7610
Ebsco	((hypomineralisation OR hypomineralization OR hypomaturation OR hypocalcification) OR (molar incisor hypomineralization OR molar incisor hypomineralisation) OR (hypomineralised permanent molars OR hypomineralized permanent molars)) AND ("restoration" OR "restorative" OR "adhesion" OR "adhesive" OR "bond")	174

Selection of Studies 

The studies were screened by a single author for their title, abstract, and full text. The titles of the articles were read in the first stage, and those studies that didn’t fulfill the inclusion criteria described above were eliminated. Any duplicates identified across the databases were removed manually. Abstracts were read in the second stage, and selection criteria were applied. If the information given in the abstract wasn’t sufficient, then the full text of the articles was read. In the third stage, the full texts of the studies that matched the PICOS format were read, and the necessary relevant data were extracted. Only studies dealing with the application of composite resin restorations or GIC on hypomineralized first permanent molars were included. An additional manual search was conducted after reviewing the reference lists of the eligible studies, resulting in the final selection of studies.

A supervisor further reviewed the articles to verify their eligibility, and any additional articles deemed irrelevant to the current review were excluded. Any disagreements about the selected studies were addressed and resolved through discussion among the reviewers. In case of dispute over the selection of studies, the study in the most recent publication was considered. The quality assessment of each article was performed by the principal investigator and was further verified by the supervisor for inclusion of articles for meta-analysis. The search finally yielded articles to be included in the systematic review. The excluded studies were documented along with the reason for their exclusion. The author wasn’t blinded to any source of information of the included studies.

*Data Extraction* 

Microsoft Excel (Microsoft Corp., Redmond, WA, USA) was used to prepare a standardized data extraction sheet and highlight the relevant findings. The collected data have been described in Tables 4-5.

**Table 4:** Data extracted for primary outcome MIH: Molar incisor hypomineralization; HT: High translucency; GIC: Glass ionomer cement; SE: Self etch; USPHS: United States Public Health Service; TNI: Treatment need index; PEB: Post Eruptive Breakdown; EAPD: European Academy of Pedaitric Dentistry; FDI: Federation Dentaire Internationale; CAD/CAM: Computer aided design/computer aided manufacturing; ART: Atraumatic restorative treatment

Sr. No.	Author (year of publication)	Type of study	Sample size (loss to follow-up)				Age group	Severity of MIH	Type of treatment				Criteria (visual-tactile)	Outcome (overall survival)				Follow-up	Annual failure rate			
			Group I	Group II	Group III	Group IV			Group I	Group II	Group III	Group IV		Group I	Group II	Group III	Group IV		Group I	Group II	Group III	Group IV
1.	Rolim et al. (2020) [7]	Randomized clinical trial	33 molars (n = 2 at 6^th^ month)	31 molars (n = 3 at 12^th^ month)	-	-	Mean age 10 years (seven to 16 years)	Moderate to severe	Total etch (Ultradent; Ultradent Products Inc., South Jordan, UT, USA) followed by Ambar Universal Adhesive (FGM Dental Group, Joinville, SC, BRA) and Tetric-N-Ceram Bulk Fill composite (Ivoclar, Schaan, LIE)	Ambar Universal Adhesive and Tetric-N-Ceram Bulk Fill composite	-	-	Modified USPHS criteria	25 molars	17 molars	-	-	12 months	19.36%	39.29%	-	-
2.	Sonmez et al. (2017) [10]	Randomized clinical trial	32 molars having MIH (n = 0)	31 molars having MIH (n = 0)	32 molars having MIH (n = 0)	31 molars (carious without MIH) (n = 0)	Eight to 12 years	Post-eruptive breakdown (PEB) associated with carious lesions	Complete hypomineralized tissue removal followed by etching (ETCH37TM; Bisco Inc., Schaumburg,IL, USA), Futurabond self-etch adhesive (VOCO GmbH, Cuxhaven, DEU) and nanohybrid composite material (Grandio; VOCO GmbH)	Selective carious and hypomineralized tissue removal followed by etching (ETCH37TM), Futurabond self-etch adhesive, and Grandio nanohybrid composite material	Selective carious and hypomineralized tissue removal followed by etching (ETCH37TM), 5% NaOCl, Futurabond self-etch adhesive and Grandio nanohybrid composite material	Control group (carious without MIH)	Modified USPHS criteria	26 molars	18 molars	25 molars	25 molars	24 months	18.75%	41.94%	21.88%	19.36%
3.	Hernandez et al. (2019) [14]	Single-arm interventional study	281 molars (n = 0)	-	-	-	Six to eight years	Severe	GIC (Equia; GC International AG, Lucerne, CHE) for six months (interim) followed by composite resin restoration (Scotchbond Multi-Purpose Adhesive and Filtek^TM^ Supreme XTE; 3M, Saint Paul, MN, USA)	-	-	-	Own criteria	272 molars	-	-	-	24 months	3.20%	-	-	-
4.	Grossi et al. (2018) [15]	Prospective cohort study	59 molars (n = 4)	-	-	-	Seven to 13 years	Teeth with PEB already involving dentin or those with atypical unsatisfactory restoration (severe MIH)	Cavity Conditioner ® (GC International AG), glass hybrid restorative system (Equia Forte®; GC International AG)	-	-	-	Modified ART criteria	54 molars	-	-	-	12 months	1.82%	-	-	-
5.	De Souza et al. (2016) [16]	Randomized clinical trial	19 molars (n = 0)	22 molars (n = 0)	-	-	Six to eight years	PEB or unsatisfactory atypical restoration with or without carious lesions	GIC (Ketac Molar EasyMix; 3M) as interim followed by self-etching adhesive (Clearfil SE Bond; Kuraray Noritake Dental Inc., Tokyo, JPN) and composite resin restoration (Filtek XT350; 3M)	GIC (Ketac Molar EasyMix) as interim followed by total-etch adhesive (Adper ScotchBond Multi-Purpose Adhesive) and composite resin restoration (Filtek XT350)	-	-	Modified USPHS criteria	13 molars	13 molars	-	-	18 months	31.56%	40.91%	-	-
6.	Sen Yavuz et al. (2024) [17]	Randomized clinical trial	31 molars (n = 1 at 36 months)	31 molars (n = 1 at 36 months)	-	-	Six to 12 years	Moderate to severe	Bulk fill glass hybrid restorative (Equia Forte HT; GC International AG)	Short fiber- reinforced composite (Ever X Flow^TM^; GC International AG) covered by micro-hybrid composite (G-ænial Posterior; GC International AG)	-	-	Modified USPHS criteria	23 molars	28 molars	-	-	36 months	23.33%	6.67%	-	-
7.	Hakmi et al. (2023) [18]	Randomized clinical trial	20 molars (n = 0)	20 molars (n = 0)	-	-	Seven to 10 years	Severe	Phosphoric acid (3M), Single Bond^TM^ (3M) and direct composite resins (Filtek 350; 3M)	Indirect composite resins	-	-	Modified USPHS criteria	17 molars	18 molars	-	-	12 months	15%	10%	-	-
8.	Durmus et al. (2020) [19]	Prospective single-arm interventional study	134 molars (n = 0)	-	-	-	Eight to 11 years	MIH TNI 2a-c	High viscosity GIC (Equia Forte®)	-	-	-	Modified USPHS criteria	117 molars	-	-	-	24 months	12.68%	-	-	-
9.	Linner et al. (2020) [20]	Retrospective cohort study	28 molars (n = 0)	127 molars (n = 0)	27 molars (n = 0)	23 molars (n = 0)	Mean age 11.2 years	Mild to moderate	GIC (Ketac Molar EasyMix)	Non-invasive composite restorations: Adper Prompt L-Pop or Scotchbond Universal L-Pop (3M) and Tetric EvoFlow (Ivoclar)	Conventional composite restorations: Etching, adhesive bonding agent (Syntac Classic; Ivoclar), composite restorations (Tetric EvoCeram; Ivoclar)	CAD/CAM-fabricated ceramic restorations: (Celtra Duo; Dentsply Sirona, Charlotte, NC, USA)	EAPD, FDI	2 molars	37 molars	21 molars	23 molars	36 months	NA	NA	NA	NA
10.	Fragelli et al. (2015) [21]	Prospective cohort study	48 molars (n = 10)	-	-	-	Six to nine years	Unsatisfactory atypical restorations and PEB, and associated with or without caries	GIC (Ketac Molar Easymix)	-	-	-	Modified USPHS criteria	35 molars	-	-	-	12 months	7.90%	-	-	-
11.	Mejare et al. (2005) [22]	Retrospective cohort study	63 molars (n = 0)	34 molars (n = 0)	-	-	Mean age 8.5 years	Mild and severe	GIC restoration	Composite restoration	-	-	Ryge criteria	31 molars	29 molars	-	-	10 years (mean)	NA	NA	NA	NA
12.	Lygidakis et al. (2003) [23]	Prospective single-arm interventional study	49 molars (n = 0)	-	-	-	Eight to 10 years	Chronological enamel hypomineralization of systemic origin, teeth having more than two surfaces involved that may or may not have been restored before	One bottle adhesive (Prime&Bond; Dentsply Sirona) followed by hybrid composite (Brilliant/Synergy; Coltene, Altstätten, CHE)	-	-	-	Cvar and Ryge criteria	49 molars	-	-	-	48 months	0% (Nil)	-	-	-
13.	Ozsoy et al. (2024) [24]	Randomized clinical trial	48 molars (n = 3 at 3^rd ^month)	49 molars (n = 4 at 3^rd^ month)	46 molars (n = 1 at 3^rd^ month)	46 molars (n = 2 at 3^rd^ month)	Eight to 15 years	Teeth with 2b, 2c, 4b, and 4c scores according to MIH TNI	Bulk fill glass hybrid restorative (Equia Forte HT)	GIC (Fuji IX; GC International AG) as base followed by Clearfil SE Bond and posterior composite (G-ænial)	Clearfil SE Bond followed by posterior composite (EverX) and posterior composite (G-ænial)	Papacarie gel (Papacarie Duo; Dentaltix, Madrid, ESP) followed by Clearfil SE Bond, posterior composite EverX and posterior composite G-ænial	Modified USPHS criteria	38 molars	41 molars	43 molars	38 molars	9 months	15.56%	8.89%	4.44%	13.64%

**Table 5:** Data extracted for secondary outcome MIH: Molar incisor hypomineralization; HT: High translucency; GIC: Glass ionomer cement; SE: Self etch; USPHS: United States Public Health Service

Sr. No.	Author (year of publication)	Type of study	Sample size				Type of treatment				Anatomical form				Marginal adaptation				Surface texture				Marginal discolouration				Retention				Secondary caries				Post-operative sensitivity				Colour match				Follow-up
			Group I	Group II	Group III	Group IV	Group I	Group II	Group III	Group IV	Group I	Group II	Group III	Group IV	Group I	Group II	Group III	Group IV	Group I	Group II	Group III	Group IV	Group I	Group II	Group III	Group IV	Group I	Group II	Group III	Group IV	Group I	Group II	Group III	Group IV	Group I	Group II	Group III	Group IV	Group I	Group II	Group III	Group IV	
1.	Rolim et al. (2020) [7]	Randomized clinical trial	33 teeth	31 teeth	-	-	Total etch (Ultradent) followed by Ambar Universal Adhesive and Tetric-N-Ceram Bulk Fill composite	Ambar Universal Adhesive and Tetric-N-Ceram Bulk Fill composite	-	-	21 (72.41%)	15 (53.57%)	-	-	22(75.86%)	15 (53.57%)	-	-	22 (75.86%)	19 (67.86%)	-	-	23 (79.81%)	20 (71.43%)	-	-	24 (82.76%)	18 (64.23%)	-	-	26 (89.66%)	22 (78.57%)	-	-	NA	NA	-	-	NA	NA	-	-	12 months
2.	Sonmez et al. (2017) [10]	Randomized clinical trial	32 molars (MIH)	31 molars (MIH)	32 molars (MIH)	31 molars (Control group without MIH)	Complete hypomineralized tissue removal followed by etching (ETCH37TM), Futurabond Self-etch Adhesive and nanohybrid composite material (Grandio)	Selective carious and hypomineralized tissue removal followed by etching (ETCH37TM), Futurabond Self-etch Adhesive and nanohybrid composite material (Grandio)	Selective carious and hypomineralized tissue removal followed by etching (ETCH37TM), 5% NaOCl, Futurabond Self-etch Adhesive and nanohybrid composite material (Grandio)	Control group (carious without MIH)	24 (75%)	17 (50%)	23 (71.88%)	26 (83.37%)	24 (75%)	17 (50%)	23 (71.88%)	26 (83.37%)	26 (81.25%)	20 (64.52%)	26 (81.25%)	28 (90.32%)	25 (78.13%)	17 (50%)	24 (75%)	27 (87.10%)	26 (81.25%)	20 (64.52%)	26 (81.25%)	28 (90.32%)	26 (81.25%)	19 (61.29%)	26 (81.25%)	28 (90.32%)	26 (81.25%)	20 (64.52%)	26 (81.25%)	28 (90.32%)	NA	NA	NA	NA	24 months
3.	De Souza et al. (2016) [16]	Randomised clinical trial	19 molars	22 molars	-	-	GIC (Ketac Molar EasyMix) as interim followed by self-etching adhesive (Clearfil SE Bond) and composite resin restoration (Filtek XT350)	GIC (Ketac Molar EasyMix) as interim followed by total-etch Adhesive (Adper ScotchBond Multi-Purpose) and composite resin restoration (Filtek XT350)	-	-	13 (68.42%)	12 (54.55%)	-	-	13 (68.42%)	12 (54.55%)	-	-	14 (73.68%)	12 (54.55%)	-	-	14 (73.68%)	12 (54.55%)	-	-	13 (68.42%)	12 (54.55%)	-	-	13 (68.42%)	13 (59.10%)	-	-	NA	NA	-	-	NA	NA	-	-	18 months
4.	Sen Yavuz et al. (2024) [17]	Randomized clinical trial	31 teeth	31 teeth	-	-	Bulk fill glass hybrid restorative (Equia Forte HT)	Short fiber- reinforced composite (EverX Flow^TM^ covered by micro-hybrid composite (G-ænial Posterior)	-	-	23 (76.67%)	27 (90%)	-	-	18 (60%)	22 (73.33%)	-	-	NA	NA	-	-	22 (73.33%)	24 (80%)	-	-	18 (60%)	22 (73.33%)	-	-	23 (76.67%)	24 (80%)	-	-	22 (73.33%)	26 (86.67%)	-	-	21 (70%)	19 (63.33%)	-	-	36 months
5.	Hakmi et al. (2023) [18]	Randomized clinical trial	20 molars	20 molars	-	-	Phosphoric acid (3M), Single Bond^TM ^and direct composite resins (Filtek 350)	Indirect composite resins	-	-	17 (85%)	19 (95%)	-	-	16 (80%)	16 (80%)	-	-	12 (60%)	17 (75%)	-	-	15 (75%)	16 (80%)	-	-	20 (100%)	20 (100%)	-	-	18 (90%)	19 (95%)	-	-	17 (85%)	19 (95%)	-	-	NA	NA	-	-	12 months
6.	Durmus et al. (2020) [19]	Prospective single-arm interventional study	134 molars	-	-	-	High viscosity GIC (Equia Forte)	-	-	-	97 (72.39%)	-	-	-	100 (74.63%0	-	-	-	109 (81.34%)	-	-	-	101 75.37%)	-	-	-	109 (81.34%)	-	-	-	115 (85.82%)	-	-	-	114 (85.07%)	-	-	-	107 (79.85%)	-	-	-	24 months
7.	Lygidakis et al. (2003) [23]	Randomised clinical trial	49 molars	-	-	-	One bottle adhesive (Prime&Bond) followed by hybrid composite (Brilliant/Synergy)	-	-	-	45 (91.84%)	-	-	-	49 (100%)	-	-	-	46 (93.88%)	-	-	-	NA	-	-	-	NA	-	-	-	49 (100%)	-	-	-	NA	-	-	-	39 (79.59%)	-	-	-	48 months
8.	Ozsoy et al. (2024) [24]	Randomized clinical trial	48 teeth	49 teeth	46 teeth	46 teeth	Bulk fill Glass hybrid restorative (Equia Forte HT)	GIC (Fuji IX) as base followed by Clearfil SE Bond and posterior composite G-ænial	Clearfil SE Bond followed by posterior composite EverX and posterior composite G-ænial	Papacarie gel (Papacarie Duo) followed by Clearfil SE Bond, posterior composite EverX and posterior composite G-ænial	NA	NA	NA	NA	29 (64.44%)	30 (66.67%)	39 (86.67%)	36 (81.82%)	30 (66.67%)	41 (91.11%)	43 (95.56%)	38 (86.37%)	38 (84.44%)	39 (86.67%)	41 (91.11%)	37 (84.10%)	38 (84.44%)	41 (91.11%)	43 (95.56%)	38 (86.37%)	38 (84.44%)	41 (91.11%)	43 (95.56%)	37 (84.10%)	NA	NA	NA	NA	36 (80%)	41 (91.11%)	42 (93.33%)	38 (86.37%)	9 months

Risk of Bias Within Studies

The methodological quality of the results extracted from the included studies and the risk of bias were evaluated by a single author and reviewed by a supervisor. The Cochrane Collaboration's tool for assessing the risk of bias was used for randomized clinical studies, and the risk of bias in non-randomized studies of intervention (ROBINS-I) tool was used for non-randomized clinical studies.

Statistical Analysis

Meta-analysis was conducted when the data’s quality and quantity warranted it. The software STATA 17.0 (StataCorp LLC, College Station, TX, USA) was utilized to analyze the random effects model, assess heterogeneity, and evaluate publication bias in order to test for the differences in success rates between the control and experimental groups. Heterogeneity was evaluated using Cochrane's Q test and I² statistics, where substantial heterogeneity was denoted when I² > 50% or a p-value < 0.10 from Cochrane's Q test was obtained. A p-value < 0.05 was considered statistically significant.

Results

Study Selection

**Figure 1:**
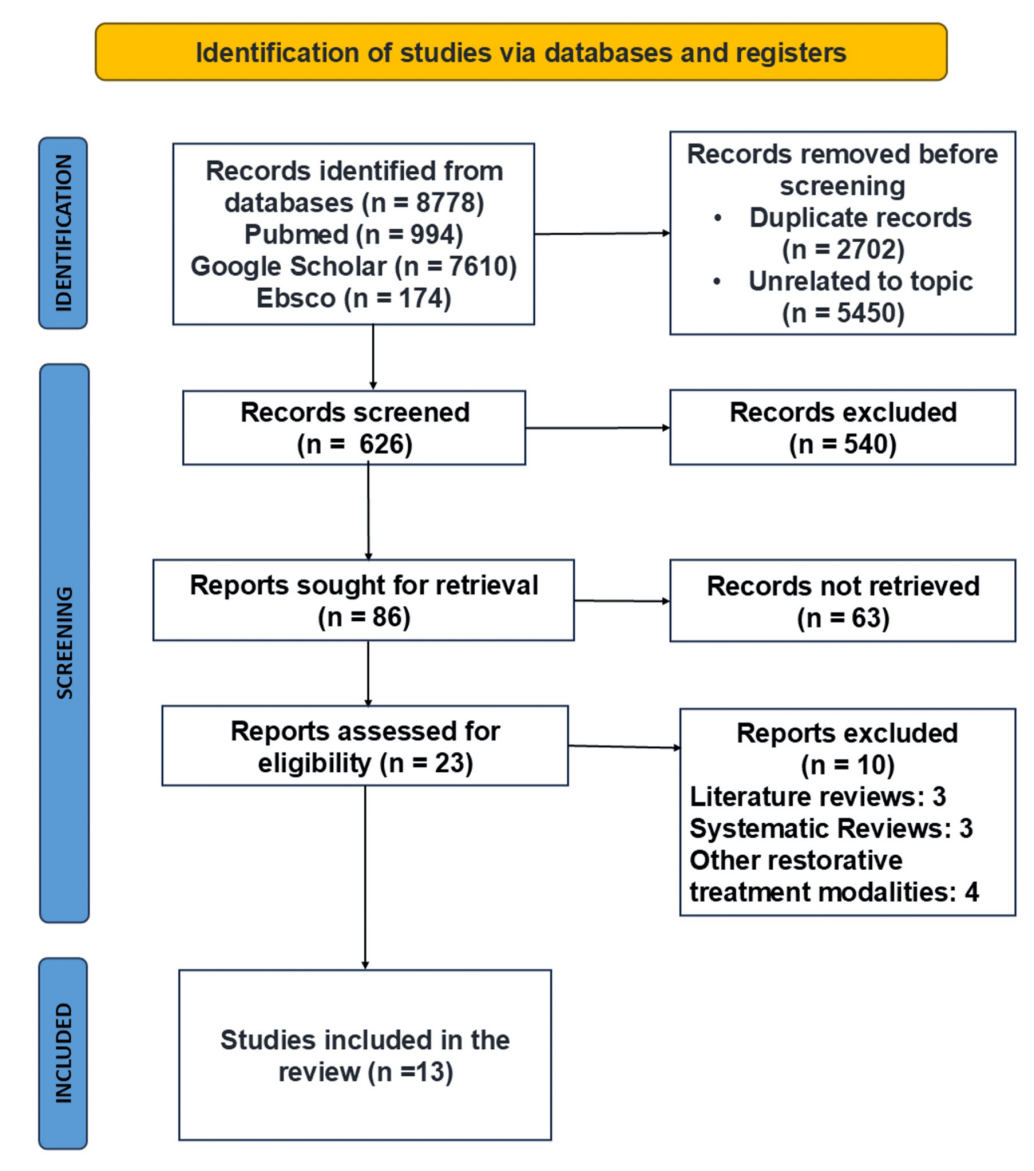
PRISMA flowchart of study selection PRISMA: Preferred Reporting Items for Systematic Reviews and Meta-Analysis This flowchart was created by author Malayka Shah.

A PRISMA flow diagram depicting the details of the search results is described in Figure 1. Thirteen articles were qualitatively analyzed and included in the systematic review. No new articles were added by screening the reference lists and manual searching.

Description of the Included Studies

The characteristics of the 13 included studies [7,10,14-24] are listed in Tables 3 -4. Six of the total identified studies were randomized clinical studies [7,10,16,17,18,24], three studies [14,19,23] were single-arm interventional studies, two were prospective cohort studies [15,21], and two were retrospective cohort studies [20,22]. One study [18] was a split-mouth study. The sample size in each study varied from 40 to 281, and the participants' ages ranged between six and 18 years. The severity of MIH was assessed using the judgment criteria for MIH in epidemiological studies [25] in six studies [7,10,15,16,21,22], the MIH treatment need index [26] in three studies [17,19,24], best practice guidelines for MIH [27] in two studies [18,20], the MIH training manual [28] in one study [14], and was described as MIH of systemic origin in one study [23].

Among the 13 studies, four evaluated both GIC and composite resin restorations [17,20,22,24], six addressed composite resin restorations [7,10,14,16,18,23], and three addressed GIC restorations [15,19,21]. For randomized studies, the follow-up duration ranged between nine and 48 months, whereas it varied from 12 months to 10 years for non-randomized clinical studies. Modified USPHS criteria were applied in 10 studies [7,10,16-19,21-24], one study [20] used FDI criteria, one study [15] used modified atraumatic restorative treatment (ART) criteria, and one study [14] used its own criteria for visual-tactile examination.

Evaluation of Success Rate

Using the modified USPHS criteria [29], the success rate was calculated for 10 studies [7, 10, 16-19, 21-24], out of which two [21,22] didn’t provide a detailed evaluation table. For studies dealing with composite resin, the success rates for anatomical form ranged from 50% [17] to 95% [10]. Whereas, for GIC restorations, it varied from 72.39% [19] to 76.67% [17]. The success rates for marginal adaptation ranged from 50% [10] to 100% [23] for studies dealing with composite resin, and for GIC restorations, it ranged from 60% [19] to 74.63% [17]. While evaluating the parameter of surface texture, the success rates ranged from 54.55% [16] to 95.56% [24] for studies dealing with composite resin. For GIC restorations, it ranged from 66.67% [24] to 81.34% [19]. The next parameter evaluated was marginal discoloration, for which the success rates ranged from 50% [10] to 91.11% [24] for the studies evaluating composite resin and from 73.33% [17] to 84.44% [24] for the studies evaluating GIC.

For retention, the success rates ranged from 54.55% [10] to 100% [17] for the studies dealing with composite resin and from 60% [17] to 84.44% [24] for studies dealing with GIC. The success rates for the parameter of secondary caries ranged from 59.10% [16] to 100% [23] for studies dealing with composite resin and ranged from 76.67% [17] to 85.82% [19] for studies dealing with GIC. While calculating the success rates for the parameter of postoperative sensitivity, the values ranged from 64.52% [10] to 95% [18] for studies dealing with composite resin and from 73.33% [17] to 85.07% [19] for studies dealing with GIC. The last parameter evaluated was color match; the success rates ranged from 63.33% [17] to 93.33% [24] for studies dealing with composite resin and from 70% [17] to 80% [24] for studies dealing with GIC. The overall annual failure rate (AFR) ranged from nil to 41.94%. The AFR for studies evaluating composite resin restorations varied from nil [23] to 41.94% [10]. For the studies dealing with GIC restorations, the AFR ranged from 1.82% [15] to 23.33% [17].

Other Parameters Assessed

Rolim et al. [7] used the Faces Pain Scale-revised [30] for the evaluation of self-reported pain before and after treatment and the Venham Picture Test (VPT) [31] for the evaluation of anxiety before and after treatment. Hakmi et al. [18] used the Faces Pain Scale [32] for the evaluation of child satisfaction by recording the state of anxiety. Durmus et al. [19] assessed the behavior of patients during treatment using the Houpt Behavior Rating Scale [33]. Ozsoy et al. [24] evaluated the demographic data of the participants to understand the etiology of MIH. Lygidakis et al. [23] reported hypersensitivity separately on a scale of two severity levels based on patients' answers.

Risk of Bias Within Studies

**Figure 2:**
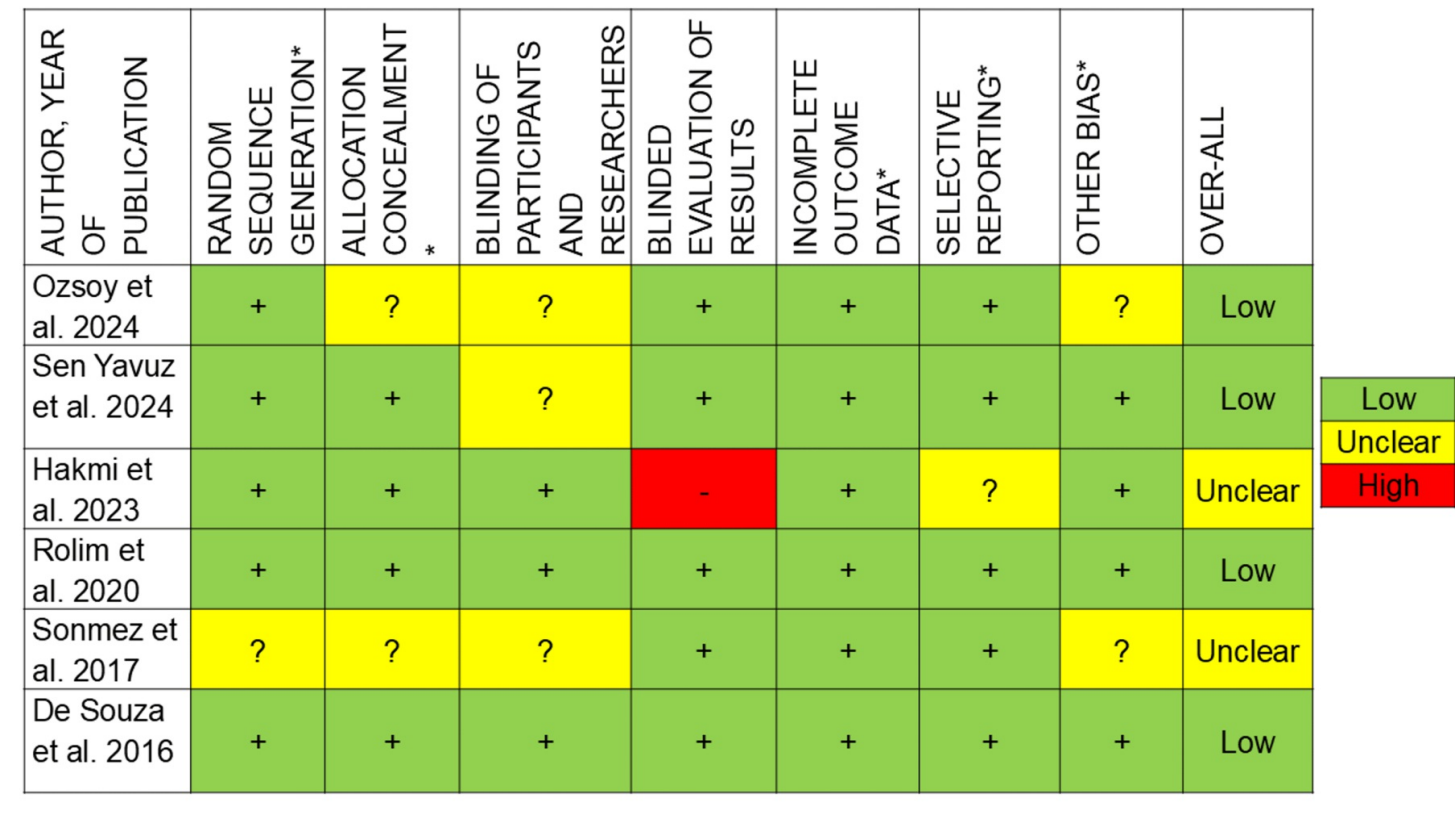
Risk of bias for randomised clinical trials Ozsoy et al. [24], Sen Yavuz et al. [17], Hakmi et al. [18], Rolim et al. [7], Sonmez et al. [10], De Souza et al. [16]. * Indicates key domains for evaluating risk of bias. This figure was created by author Malayka Shah.

**Figure 3:**
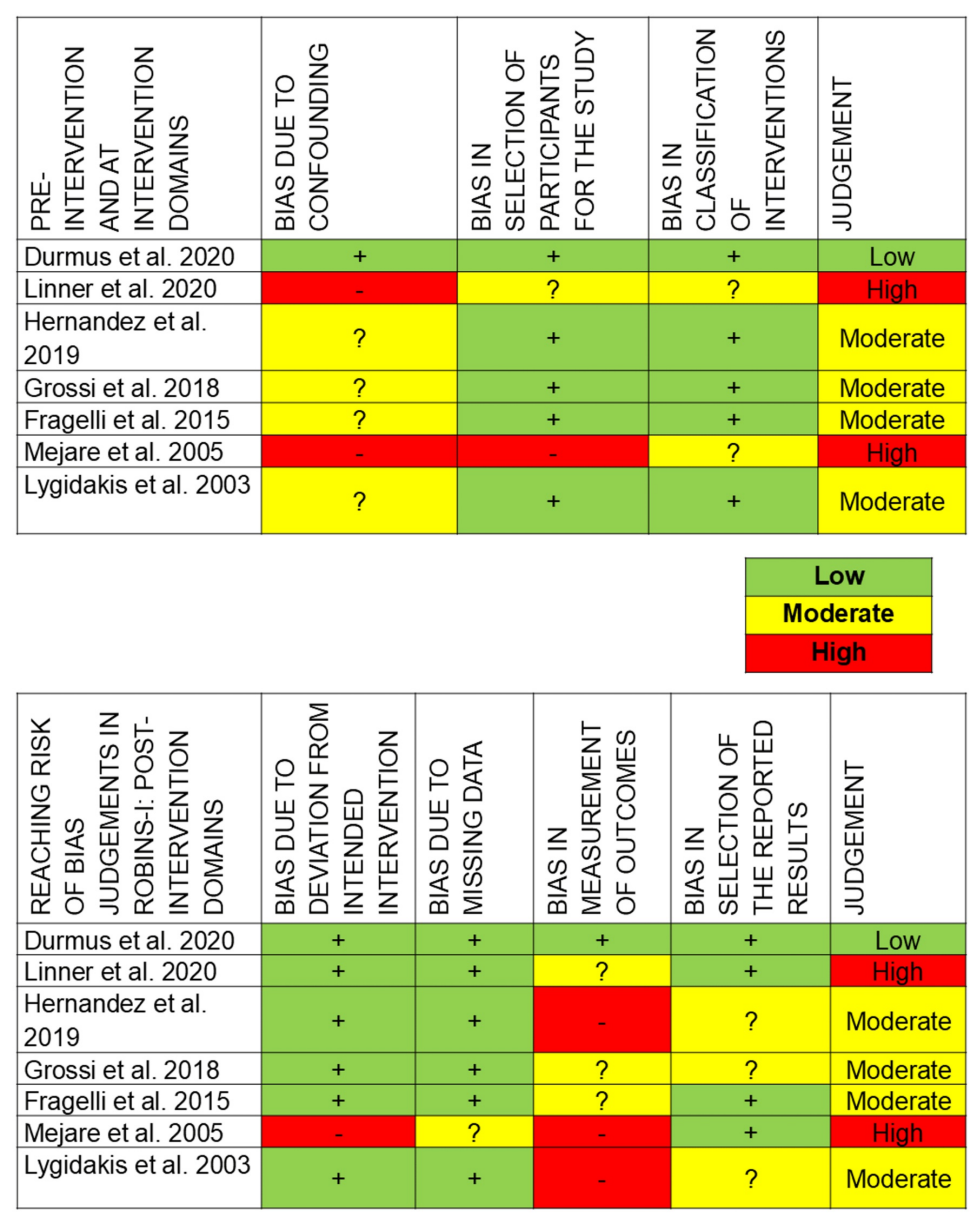
Risk of bias for non-randomised clinical trials Durmus et al. [19], Linner et al. [20], Hernandez et al. [14], Grossi et al. [15], Fragelli et al. [21], Mejare et al. [22], Lygidakis et al. [23]. This figure was created by author Malayka Shah.

The results of the risk of bias are described in Figures 2-3. Selection bias on the basis of allocation concealment was recorded as unclear for Ozsoy et al. [24], Sonmez et al. [10], and Linner et al. [20] as sufficient information wasn’t provided. Confounding bias was considered moderate by Hernandez et al. [14], Grossi et al. [15], Fragelli et al. [21], and Lygidakis et al. [23], where other factors might have influenced the results. Detection bias was denoted as high risk in Hernandez et al. [14], Mejare et al. [22], and Lygidakis et al. [23], where the blinded evaluation of results wasn’t considered, as it would affect the results and follow-ups. Reporting bias was considered unclear in Hakmi et al. [18] and of moderate risk in Hernandez et al. [14], Grossi et al. [15], and Lygidakis et al. [23], where the examiner wasn’t blinded and would affect the results. Other bias was scored as unclear in Ozsoy et al. [24] and Sonmez et al. [10], where factors such as patient compliance could have affected the results.

Among the 13 studies, Linner et al. [20] and Mejare et al. [22] reported having a high risk of bias. Hakmi et al. [18] and Sonmez et al. [10] reported having an unclear risk of bias. Hernandez et al. [14], Grossi et al. [15], Fragelli et al. [21], and Lygidakis et al. [23] reported having moderate risk of bias. Ozsoy et al. [24], Sen Yavuz et al. [17], Rolim et al. [7], De Souza et al. [16], and Durmus et al. [19] reported having a low risk of bias.

Synthesis of results

Meta-Analysis

In the meta-analysis, only the studies that evaluated both GIC and composite resin restorations for hypomineralized first permanent molars with available data for each parameter analyzed were included, so that the meta-analysis with different numbers of studies was presented. For studies that reported the use of multiple composite resins, all resins were considered. The number of samples showing success at the final follow-up period was considered out of the total sample size. The results obtained were shown over the forest plot and funnel plot while considering a 95% confidence interval. A random-effects model was fitted to the data. The amount of heterogeneity, i.e., tau², was estimated using the restricted maximum-likelihood estimator [34]. The Q-test for heterogeneity [35] and the I² statistics were also reported. The Cochran Q test was employed to determine heterogeneity between studies. The I² test was used to determine the proportion of inconsistency in the pooled estimates attributable to between-study heterogeneity.

Meta-Analysis for Overall Success

**Figure 4:**
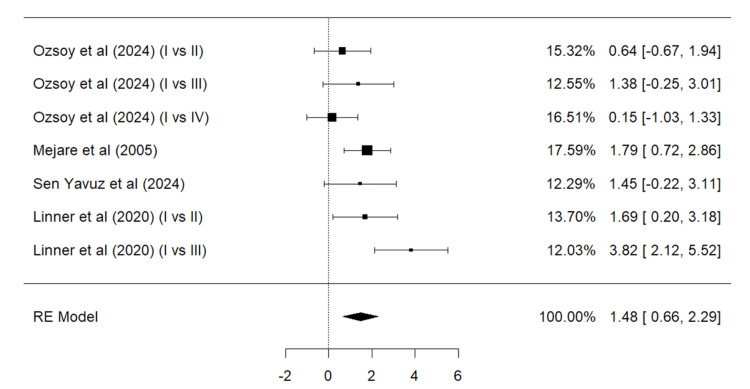
Forest plot (I) on the overall success of GIC vs. composite resin Ozsoy et al. [24],  Mejare et al. [22], Sen Yavuz et al. [17], Linner et al. [20] RE: Random effect, GIC: Glass ionomer cement This igraph was created by author Malayka Shah.

A total of four studies (Ozsoy et al. [24], Sen Yavuz et al. [17], Linner et al. [20], and Mejare et al. [22]) were included in the meta-analysis (Figure 4). The results for overall success were significantly superior for composite resin restorations than GIC (p = 0.0004). According to the Q test, the true outcomes appear to be moderately heterogeneous (Q = 14.0242, p = 0.0294, tau² = 0.6845, I² = 57.7228%). Neither the rank correlation nor the regression test indicated any funnel plot asymmetry (p = 0.3813 and p = 0.1902, respectively).

Meta-Analysis for Parameters of the Modified USPHS Criteria

**Figure 5:**
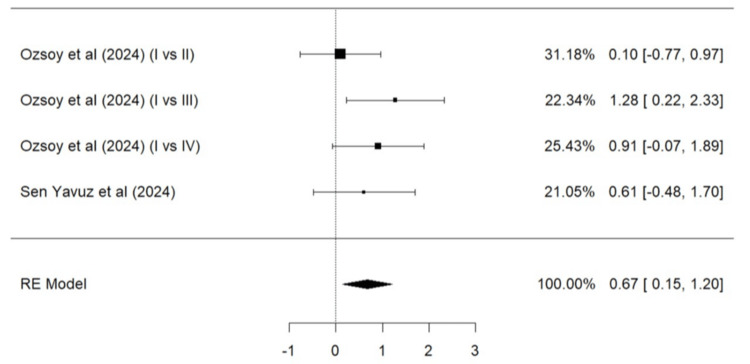
Forest Plot (II) on successful outcome of marginal adaptation (GIC vs. composite resin) Ozsoy et al. [24], Sen Yavuz et al. [17] RE: Random effect, GIC: Glass ionomer cement This graph was created by author Malayka Shah.

**Figure 6:**
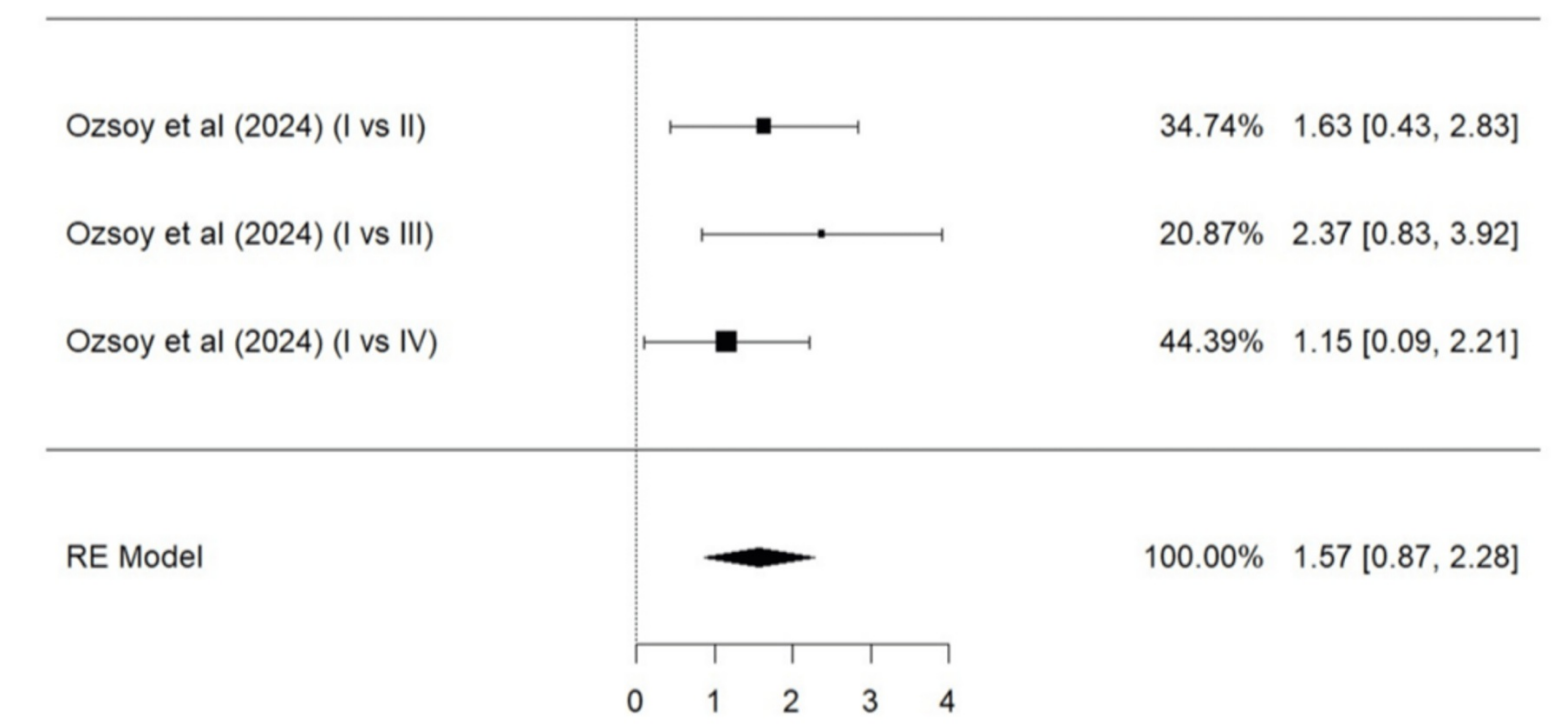
Forest plot (III) on successful outcome of surface texture (GIC vs. composite resin) Ozsoy et al. [24] RE: Random effect, GIC: Glass ionomer cement This graph was created by author Malayka Shah.

**Figure 7:**
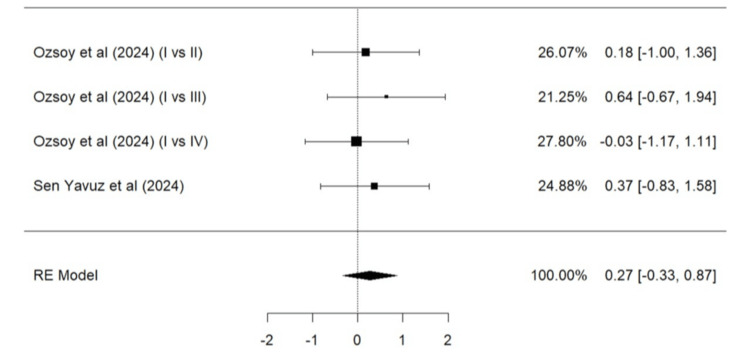
Forest plot (IV) on success of marginal discoloration (GIC vs. composite resin) Ozsoy et al. [24], Sen Yavuz et al. [17] RE: Random effect, GIC: Glass ionomer cement This graph was created by author Malayka Shah.

**Figure 8:**
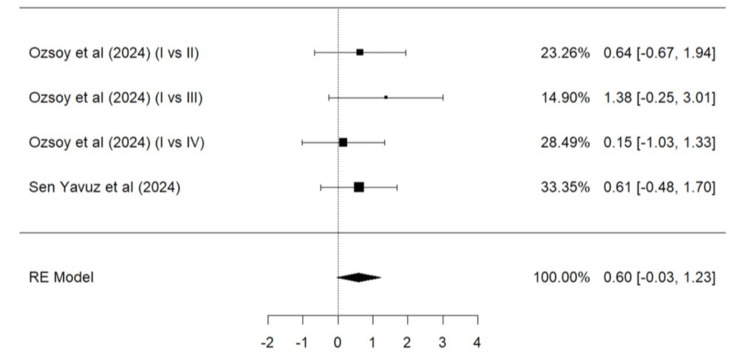
Forest plot (V) on success of retention (GIC vs. composite resin) Ozsoy et al. [24], Sen Yavuz et al. [17] RE: Random effect, GIC: Glass ionomer cement This graph was created by author Malayka Shah.

**Figure 9:**
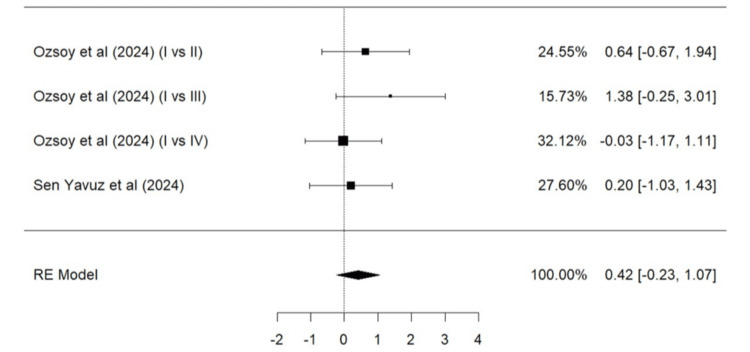
Forest plot (VI) on success of secondary caries (GIC vs. composite resin) Ozsoy et al. [24], Sen Yavuz et al. [17] RE: Random effect, GIC: Glass ionomer cement This graph was created by author Malayka Shah.

**Figure 10:**
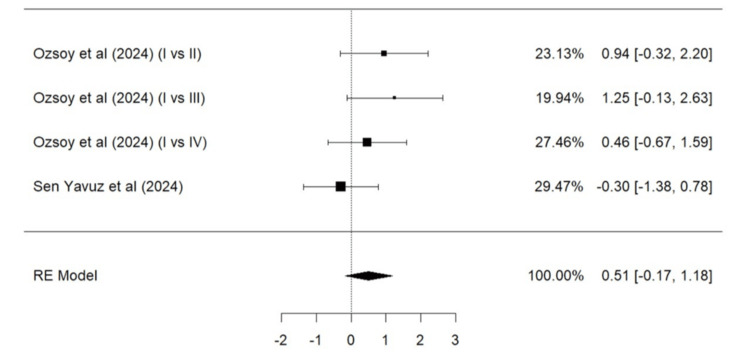
Forest plot (VII) on the success of colour match (GIC vs. composite resin) Ozsoy et al. [24], Sen Yavuz et al. [17] RE: Random effect, GIC: Glass ionomer cement This graph was created by author Malayka Shah.

Parameters such as marginal adaptation, surface texture, marginal discoloration, retention, secondary caries, and color match based on the modified USPHS criteria were evaluated. A total of two studies (Ozsoy et al. [24] and Sen Yavuz et al. [17]) were included in the meta-analysis for marginal adaptation (Figure 5), marginal discoloration (Figure 7), retention (Figure 8), secondary caries (Figure 9), and color match (Figure 10). One study, Ozsoy et al. [24], was included in the meta-analysis for surface texture (Figure 6). Parameters such as marginal adaptation and surface texture gave statistically significant superior results in favor of composite resin over GIC (p = 0.01 and p<0.0001, respectively). Parameters of marginal discoloration, retention, secondary caries, and color match also gave superior results in favor of composite resin but were statistically non-significant (p = 0.3, p = 0.06, p = 0.2, and p = 0.14, respectively).

Discussion

Molar incisor hypomineralization has a direct influence on the oral health-related quality of life (OHRQoL) of children. With an increase in severity, a more severe influence can be seen [36]. Cabral et al. state that in teeth with a post-eruptive breakdown (PEB), the occlusal surface collapses, leaving the dentin exposed to increased clinical signs such as pain and hypersensitivity [37]. Molar incisor hypomineralization commonly presents with tooth hypersensitivity in children. The increased sensitivity leads to the deterioration of oral hygiene with increased plaque accumulation, resulting in higher susceptibility to dental caries [38]. The teeth that are severely affected require an early restorative intervention. Dental behavior management problems are also common in children having MIH because of repeated experiences of pain and discomfort [5]. Thus, the choice of restorative material and technique must consider the severity and extent of the defect, the quality of the affected and sound enamel, the presence of hypersensitivity, the age of the patient, and the level of cooperation [39].

The restorative treatment modalities are performed with the aim of restoring the anatomic form and function of the affected teeth. Several systematic reviews have been published that summarize all the restorative treatment options available for MIH-affected teeth. Glass ionomer cement and composite resin are the usual choices of materials from the standpoint of minimally invasive dentistry. However, there is a gap in the literature regarding the success rate of GIC and composite resin due to conflicting evidence from different studies.

A previous systematic review by Lopes-Fatturi et al. assessed the restorative treatment modalities for MIH-affected teeth, and it was concluded that variability of restorative techniques and materials exists, and direct restorative options like GIC and composite resins could be the first choices for restoration in permanent first molars. There’s no evidence to guide clinicians on the most favorable approaches to restoring permanent first molars affected by MIH [40]. Hypomineralized enamel has a weaker structure and thus shows poor bonding to either of the restorative materials as compared to normal enamel. Weber et al. assessed the restorations of teeth affected by MIH and concluded that clinical studies about restorations of teeth affected by MIH are very heterogeneous. Resin-modified GIC gave superior results compared to GIC, while resin composites were suitable for restoring all severities of MIH, and lower adhesion to MIH-affected enamel was seen as compared to sound enamel [41].

In our study, the results of the qualitative synthesis showed that the highest annual failure rate was shown by Group II of Sonmez et al.'s study [10] using composite resin. This could be attributed to the selective removal of the hypomineralized tissue and the use of self-etch adhesive. Better overall success was obtained when the methodology comprised complete removal of the carious or hypomineralized enamel with the cavity margins ending in sound enamel [10,18,23,24].

While measuring the overall success for the group GIC vs. composite resin, it can be seen that the results are statistically significant; the overall effect as well as all the individual studies lie in favor of the use of composite resin over GIC. Better results of composite resin over GIC can also be attributed to the different bonding strategies used with composite resin restorations. Though GIC can be the treatment of choice in teeth with adhesion difficulties, its mechanical properties and longevity of the restorative material pose certain disadvantages [42].

The results for the evaluation of secondary outcomes gave statistically significant superior findings in favor of composite resin restoration for parameters such as marginal adaptation and surface texture. The property of marginal adaptation is attributed to producing a good marginal seal for any restorative material. The different adhesion mechanisms of the material play a role in the marginal seal. Conventional GIC bonds by chemical adhesion with the tooth structure. Modified GIC tends to form open margins and fractured restoration edges when viewed under a scanning electron microscope (SEM). The application of a cavity conditioner can eliminate the formation of these microcracks [43]. Neither Sen Yavuz et al. [17] nor Ozsoy et al. [24] mention using a cavity conditioner before the use of modified GIC, which could have contributed to the significant inferiority of GIC over composite resin based on marginal adaptation. Composite resins have a smaller particle size and fillers, which are responsible for a smoother and more homogenous surface as compared to GIC. Moreover, GIC has less wear resistance to mechanical forces on long-term evaluation, which can result in poor surface texture of GIC [44]. Modified GICs used in the current study have an additional step of resin coat application that helps in increasing the wear resistance [45]. But despite that, the basic composition of glass ionomer plays a role in the overall surface texture of modified GIC, making it significantly inferior to composite resin.

Other parameters such as marginal discoloration, retention, secondary caries, and color match also gave superior findings in favor of composite resin but were statistically nonsignificant. Marginal discoloration and color match depend on the marginal seal produced by that material. Modified GIC produces less marginal discoloration and a superior color match than conventional GIC due to the application of a separate resin coat. However, their inferior marginal adaptation leads to superior findings of composite resin. Retention of any restorative material is not only associated with adhesion but also with properties like adaptation, cavity size, shape, and number of involved surfaces. Resin coat application over the modified GIC also provides micro-mechanical retention apart from chemical adhesion, which could be responsible for the non-significant results. Properties of GIC, like biocompatibility, fluoride release, and recharge, along with its coefficient of thermal expansion being similar to dentin, render it advantageous for the prevention of secondary caries. With modified GIC, the application of a resin coat results in less dissolution of the superficial layer of immature GIC and is responsible for the reduction in fluoride release [46]. Whereas composite resins don't show the formation of secondary caries until there is appropriate marginal adaptation [47]. Composite resin already had superior marginal adaptation in our review, which would have resulted in the absence of gaps at the restoration-tooth interface. Thus, leading to statistically nonsignificant but superior results of composite resin for secondary caries.

Limitations

The meta-analysis comprised only two randomized studies; thus, results can’t be generalized. More randomized studies evaluating the clinical and radiographic success of GIC and composite resin restorations in hypomineralized first permanent molars are needed. There was variability in the follow-up durations across the included studies. Evaluating the results on the basis of techniques of isolation was not within the scope of the study.

## Conclusions

For a conservative treatment approach towards hypomineralized first permanent molars, it can be concluded that composite resin restorations are the choice of restorative material within the limitations of this study. Complete removal of the hypomineralized or carious tissue is recommended over selective removal. Keeping the preparation margins in sound enamel enhances bonding but at the same time results in greater loss of tooth structure. If a selective removal approach is used, GIC can be considered as an effective option, as it bonds well with the tooth structure. Interim restoration with GIC or its use as dentin replacement can also be an efficient approach when a selective removal technique is used. Rubber dam isolation and total etch technique are recommended while using composite resin restorations, whereas a cavity conditioner is recommended while using GIC.
